# External validation of EuroSCORE II, additive EuroSCORE, and the STS predicted risk of mortality at 2,600 meters above sea level: a retrospective cohort study from a Latin American cardiac surgery centre

**DOI:** 10.3389/fcvm.2026.1840998

**Published:** 2026-10-07

**Authors:** David Rene Rodríguez-Lima, Javier Darío Garzón-Rodríguez, Paula Liliana Torres-Gómez, Cristhian Rubio-Ramos, Leonardo Andrés Gómez-Cortes, Esther María Campo-Bautista, Edith Eliana Rodriguez-Aparicio, Juan Diego Toro-Egas

**Affiliations:** 1 Critical and Intensive Care Medicine, Hospital Universitario Mayor-Méderi, Bogotá, Colombia; 2 Facultad de Medicina, Escuela de Medicina y Ciencias de la Salud, Universidad del Rosario, Bogotá, Colombia; 3 Cardiovascular Surgery, Hospital Universitario Mayor-Méderi, Bogotá, Colombia; 4 Cardiology Department, La Cardio, Bogotá, Colombia

**Keywords:** calibration, cardiac surgery, Colombia, EuroSCORE II, external validation, high altitude, risk prediction, STS PROM

## Abstract

**Background:**

EuroSCORE II, the Additive EuroSCORE, and the Society of Thoracic Surgeons Predicted Risk of Mortality (STS PROM) are widely used cardiac surgery risk models, but their performance has not been evaluated in a cardiac surgery centre located at high altitude (≥2,500 m asl).

**Methods:**

We conducted a retrospective external validation study using prospectively collected registry data from consecutive adult cardiac surgery procedures performed at Hospital Universitario Mayor-Méderi (2,600 m asl), Bogotá, Colombia, between January 2019 and February 2026 (*N* = 1,486). EuroSCORE II and the Additive EuroSCORE were calculated from the registry with the published models and evaluated in the full cohort; STS PROM was obtained from the official STS online calculator (2018 models) for the procedures within its scope (*n* = 1,076). The outcome was in-hospital mortality. Discrimination was assessed with the area under the receiver operating characteristic curve (AUC), and calibration with the observed to expected (O/E) ratio, calibration in the large, calibration slope, and calibration plots.

**Results:**

In-hospital mortality was 7.9% (117/1,486), and 82.3% of procedures were non-elective. The AUC was 0.736 (95% CI 0.687 to 0.785) for EuroSCORE II, 0.733 (0.684 to 0.783) for the Additive EuroSCORE, and 0.815 (0.757 to 0.873) for STS PROM. All three models underestimated mortality: the O/E ratio was 2.50 (2.09 to 3.00) for EuroSCORE II, 1.50 (1.25 to 1.79) for the Additive EuroSCORE, and 2.72 (2.14 to 3.47) for STS PROM. Calibration slopes were 1.04 (0.82 to 1.26), 1.94 (1.46 to 2.41), and 1.31 (1.01 to 1.60), respectively. Underestimation was present across procedure types, risk strata, and calendar years, and persisted in sensitivity analyses of variable mapping and missing data (EuroSCORE II O/E 2.37 to 3.05).

**Conclusions:**

In this high-altitude Colombian centre, EuroSCORE II, the Additive EuroSCORE, and STS PROM ranked patients acceptably, STS PROM best, but all underestimated in-hospital mortality, EuroSCORE II and STS PROM by more than twofold. The study design cannot separate an effect of altitude from differences in case mix, urgency, and local care. Local recalibration is needed before these models are used for counselling or benchmarking in this setting.

## 1 Introduction

Risk stratification is a cornerstone of contemporary cardiac surgery, providing the framework for informed clinical decision-making, surgical planning, and institutional quality assessment and benchmarking in different contexts (1, 2). Over the past decades, several prognostic models have been developed to estimate perioperative mortality, with the additive EuroSCORE (3), EuroSCORE II (4), and the Society of Thoracic Surgeons (STS) score and its latest versions (5–7) emerging as the most widely adopted tools globally.

However, despite their widespread use, the predictive accuracy of these models is often inconsistent when applied to populations that differ significantly from the original validation cohorts (8). This variability is primarily driven by heterogeneities in patient demographics, the prevalence of specific comorbidities, and the evolving complexity of surgical techniques (4, 9–14).

In Latin America this variability may be greater, and Andean populations living at high altitude may differ further because of physiological adaptation to chronic hypoxia (15, 16). Several validation studies of these models in the region have shown poor predictive performance (10, 12), yet the models remain the standard for estimating surgical risk in high-complexity centres, and, to our knowledge, none has been externally validated in a high-altitude setting. Their performance therefore needs to be evaluated in Colombian cardiac surgery centres located at high altitude.

We present, to our knowledge, the first external validation of EuroSCORE II, the additive EuroSCORE, and STS PROM in a Colombian cardiac surgery centre located at high altitude, with the aim of characterising their discrimination, calibration, and systematic prediction bias in this population.

## 2 Methods

### 2.1 Study design and setting

We conducted a retrospective cohort study of all adult patients (≥18 years) who underwent cardiac surgery at Hospital Universitario Mayor-Méderi, a 900-bed tertiary referral centre located at 2,600 m asl in Bogotá, Colombia, between 1 January 2019 and 28 February 2026. Procedures performed in January and February 2026 (*n* = 16) were included because in-hospital follow-up was complete for all of them. One record was excluded because of a data entry error (age recorded as 0). The final analytic cohort comprised 1,486 procedures. The registry does not record each patient's place of residence; however, the hospital is a referral centre for Bogotá and nearby towns at a similar altitude, and at least 97% of its patients are long-term residents of this high-altitude region. The study was approved by the ethics committee of Universidad del Rosario (DVO005 2333-CV1731) and is reported according to the TRIPOD statement (17). Patient confidentiality was maintained through sequential anonymous identifiers.

### 2.2 Study population

All consecutive adult cardiac surgery procedures were included; there were no exclusion criteria other than age below 18 years and missing outcome data. EuroSCORE II and the additive EuroSCORE were evaluated in the full cohort. STS PROM was evaluated only in procedures covered by the STS 2018 operative mortality models, namely isolated CABG, isolated aortic valve replacement (AVR), isolated mitral valve replacement (MVR), AVR plus CABG, MVR plus CABG, and mitral valve repair with or without CABG (5, 7). A procedure was assigned to one of these categories when the registry recorded exactly one of them, no concomitant procedure outside the STS models (for example, thoracic aortic surgery, surgical ablation, septal defect closure, or tumour resection), and a EuroSCORE II weight of intervention consistent with that procedure. This yielded 1,076 procedures: 757 isolated CABG, 186 isolated AVR, 54 isolated MVR, 51 AVR plus CABG, 26 MVR plus CABG, and 2 mitral valve repairs. Combined AVR plus MVR (*n* = 9) was not evaluated, because it is covered by a separate STS multiple valve model (18) rather than by the 2018 models. The reasons why the remaining 410 procedures were outside the STS scope are listed in Supplementary Table S5.

### 2.3 Data collection

Data were extracted from the institutional cardiac surgery registry (REDCap), which prospectively captures demographics, preoperative risk factors, operative characteristics, and in-hospital outcomes according to a structured data dictionary. The primary outcome was all-cause in-hospital mortality during the index admission. This outcome differs from the STS operative mortality endpoint, which also includes deaths within 30 days after surgery that occur after discharge (7). Such deaths were not captured by the registry; including them could only increase the observed mortality, so the O/E ratio for STS PROM may, if anything, be underestimated.

### 2.4 Variable definitions

Procedures were described in two ways: by procedure type, using a hierarchical and mutually exclusive classification (CABG, isolated or combined with other procedures; aortic valve surgery without CABG; mitral valve surgery without CABG; thoracic aortic surgery; and other procedures), and by the EuroSCORE II weight of intervention (isolated CABG, single non-CABG procedure, two procedures, and three or more procedures). Admission urgency was recorded according to the institutional registry, whose categories were mapped to the EuroSCORE II urgency categories: elective (scheduled admission for surgery), urgent (patients already hospitalised before surgery who could not be discharged without it), emergency (surgery required before the next working day), and salvage (cardiopulmonary resuscitation en route to or in the operating room). Under this classification, 81.0% of procedures were urgent (*n* = 1,204), 17.7% elective (*n* = 263), 1.2% emergency (*n* = 18), and 0.1% salvage (*n* = 1); combined, 82.3% were non-elective.

Each EuroSCORE II item was mapped to a registry field (Supplementary Table S1). Renal function was classified as in the original model: patients on dialysis were assigned to the dialysis category, and in the remaining patients creatinine clearance was estimated with the Cockcroft-Gault equation from preoperative serum creatinine, age, weight, and sex (19) and classified as 50 mL/min or less, 51 to 85 mL/min, or over 85 mL/min. Of the 152 patients (10.2%) with a diagnosis of chronic kidney disease, 49 were on dialysis. Creatinine clearance could not be calculated in 102 patients (6.9%) because creatinine (*n* = 91) or weight (*n* = 12) was missing; in the primary analysis these patients, apart from one on dialysis, were assigned to the normal renal function category. Left ventricular function was categorised from the preoperative echocardiographic ejection fraction with the EuroSCORE II thresholds (good, 51% or more; moderate, 31% to 50%; poor, 21% to 30%; very poor, 20% or less), and the 43 patients (2.9%) without a recorded ejection fraction were assigned to the good category. Pulmonary hypertension was categorised from the echocardiographic pulmonary artery systolic pressure (moderate, 31 to 55 mmHg; severe, over 55 mmHg) and considered absent when this pressure had not been measured (*n* = 896, 60.3%). Recent myocardial infarction was defined as infarction within 90 days before surgery. The influence of these decisions was examined in sensitivity analyses.

### 2.5 Score calculation

EuroSCORE II was calculated with the published logistic equation and coefficients, including all 18 risk factors (constant −5.324537; the coefficient for surgery on the thoracic aorta, 0.6527205, is added to the weight of intervention term) (4). The additive EuroSCORE was calculated with the original point system (3), and the point total was read as the predicted percentage mortality, as intended by its developers. STS PROM was obtained for each eligible procedure from the official STS Adult Cardiac Surgery Database Operative Risk Calculator, which implements the 2018 risk models (5, 7), queried in batch in September 2026 with an open-source client (20). Registry variables were mapped to the calculator inputs as described in Supplementary Table S2; all patients were entered as Hispanic with a non-U.S. payer, and no patient identifiers or dates were transmitted.

### 2.6 Statistical analysis

Continuous variables are reported as median and interquartile range (IQR) and were compared with the Mann–Whitney *U*-test; categorical variables are reported as counts and percentages and were compared with the chi-square or Fisher exact test. Discrimination was quantified by the AUC with 95% confidence intervals (CI) from the DeLong method, checked with 1,000 bootstrap replicates (seed 42), and AUCs of different scores in the same patients were compared with the DeLong test (21). Calibration was assessed following current recommendations (22): the O/E ratio, with a 95% CI based on the Poisson distribution of observed deaths; calibration in the large (CITL), estimated as the intercept of a logistic model with the linear predictor of the score as an offset (ideal value 0; positive values indicate underestimation); the calibration slope, estimated by regressing the outcome on the linear predictor (ideal value 1; values below 1 indicate predictions that are too extreme and values above 1 predictions that are too moderate); calibration plots by deciles of predicted risk with a loess smoother; the Brier score; and the integrated calibration index (ICI) (23). The Hosmer-Lemeshow test (10 groups) is reported for comparability with earlier studies but was not used to judge calibration, because its result depends strongly on sample size. For the additive EuroSCORE, the linear predictor was the logit of the point total divided by 100, with totals of 0 set to 0.5% (*n* = 21). O/E ratios were also calculated by procedure type, STS procedure category, EuroSCORE II weight of intervention, EuroSCORE II risk stratum, urgency, and calendar year. Subgroup AUCs were estimated only when there were at least 10 deaths and 10 survivors, and estimates based on fewer than 15 deaths are interpreted with caution. Heterogeneity of O/E ratios across procedure types and the trend across calendar years were tested with Poisson regression using the logarithm of expected deaths as an offset.

Sensitivity analyses for EuroSCORE II examined an alternative renal mapping (any chronic kidney disease assigned the coefficient for creatinine clearance of 50 mL/min or less) and the creatinine clearance category recorded in the registry; missing creatinine clearance set to 51 to 85 mL/min or to 50 mL/min or less; missing ejection fraction assigned to the moderate category; multiple imputation of creatinine, weight, and ejection fraction by chained equations (20 imputations); complete cases; alternative registry definitions of extracardiac arteriopathy, poor mobility, and critical preoperative state; and exclusion of the 2026 procedures. For STS PROM, we repeated the analysis without the 2026 procedures and in isolated CABG only. We also assessed the precision attainable with our sample size for the O/E ratio, calibration slope, and AUC (24), and performed a temporal validation in which EuroSCORE II was updated in 2019 to 2021 (intercept only, logistic recalibration, and re-estimation of all coefficients with ridge penalisation) and tested in 2022 to 2026 (25) (Supplementary Material).

Analyses were performed in Python 3.11 (scikit-learn, statsmodels, scipy, numpy, and pandas). The de-identified dataset, the STS calculator input and output files, and the analysis code are publicly available (Zenodo, DOI: https://doi.org/10.5281/zenodo.22797059).

## 3 Results

### 3.1 Study population

After exclusions, 1,486 procedures were analysed, and in-hospital mortality was 7.9% (117/1,486). Median age was 66 years (IQR 58 to 72), and 29.9% of patients were female. Most procedures were urgent (81.0%, *n* = 1,204); 17.7% were elective (*n* = 263), 1.2% emergency (*n* = 18), and 0.1% salvage (*n* = 1). CABG, isolated or combined, was the most frequent procedure (62.7%, *n* = 932), followed by aortic valve surgery without CABG (22.3%, *n* = 331), mitral valve surgery without CABG (6.6%, *n* = 98), thoracic aortic surgery (3.6%, *n* = 53), and other procedures (4.8%, *n* = 72). By EuroSCORE II weight of intervention, 53.0% of procedures were isolated CABG (*n* = 787), 27.6% single non-CABG procedures (*n* = 410), 15.7% two procedures (*n* = 234), and 3.7% three or more procedures (*n* = 55). Chronic kidney disease was recorded in 152 patients (10.2%), 49 of them on dialysis; cardiogenic shock was present in 5.4% (*n* = 80) and active endocarditis in 3.0% (*n* = 44). Baseline characteristics by survival status are shown in Table 1.

**Table 1:** Baseline characteristics of the study population by in-hospital survival.

Variable	Total (*N* = 1,486)	Survivors (*n* = 1,369)	Deaths (*n* = 117)	*p*
Age (years), median [IQR]	66 [58 to 72]	66 [58 to 72]	70 [60 to 76]	0.003
Female sex	445 (29.9%)	388 (28.3%)	57 (48.7%)	<0.001
Weight (kg), median [IQR]	68 [60 to 76]	69 [60 to 76]	67 [60 to 74]	0.193
Urgency				
Elective	263 (17.7%)	255 (18.6%)	8 (6.8%)	0.002
Urgent	1,204 (81.0%)	1,101 (80.4%)	103 (88.0%)	0.058
Emergency	18 (1.2%)	12 (0.9%)	6 (5.1%)	<0.001
Salvage	1 (0.1%)	1 (0.1%)	0 (0.0%)	1.000
Procedure type				
CABG, isolated or combined	932 (62.7%)	875 (63.9%)	57 (48.7%)	0.002
Aortic valve surgery without CABG	331 (22.3%)	304 (22.2%)	27 (23.1%)	0.919
Mitral valve surgery without CABG	98 (6.6%)	84 (6.1%)	14 (12.0%)	0.025
Thoracic aortic surgery	53 (3.6%)	43 (3.1%)	10 (8.5%)	0.006
Other procedures	72 (4.8%)	63 (4.6%)	9 (7.7%)	0.204
EuroSCORE II weight of intervention				
Isolated CABG	787 (53.0%)	749 (54.7%)	38 (32.5%)	<0.001
Single non-CABG procedure	410 (27.6%)	374 (27.3%)	36 (30.8%)	0.488
Two procedures	234 (15.7%)	201 (14.7%)	33 (28.2%)	<0.001
Three or more procedures	55 (3.7%)	45 (3.3%)	10 (8.5%)	0.008
Surgery on the thoracic aorta (EuroSCORE II item)	165 (11.1%)	142 (10.4%)	23 (19.7%)	0.004
Renal function				
Chronic kidney disease, not on dialysis	103 (6.9%)	90 (6.6%)	13 (11.1%)	0.096
Dialysis	49 (3.3%)	31 (2.3%)	18 (15.4%)	<0.001
Creatinine clearance 50 mL/min or less^a^	193 (13.0%)	173 (12.6%)	20 (17.1%)	0.217
Creatinine clearance 51 to 85 mL/min^a^	736 (49.5%)	685 (50.0%)	51 (43.6%)	0.214
Creatinine not recorded	91 (6.1%)	84 (6.1%)	7 (6.0%)	1.000
Creatinine (mg/dL), median [IQR]	0.95 [0.80 to 1.12]	0.94 [0.80 to 1.11]	1.02 [0.83 to 1.34]	0.003
Comorbidities and cardiac status				
Cardiogenic shock^b^	80 (5.4%)	36 (2.6%)	44 (37.6%)	<0.001
Active endocarditis	44 (3.0%)	32 (2.3%)	12 (10.3%)	<0.001
Atrial fibrillation or flutter	114 (7.7%)	91 (6.6%)	23 (19.7%)	<0.001
Critical preoperative state	47 (3.2%)	34 (2.5%)	13 (11.1%)	<0.001
Previous cardiac surgery	37 (2.5%)	26 (1.9%)	11 (9.4%)	<0.001
Chronic lung disease	67 (4.5%)	57 (4.2%)	10 (8.5%)	0.050
Extracardiac arteriopathy	59 (4.0%)	55 (4.0%)	4 (3.4%)	1.000
Poor mobility	10 (0.7%)	10 (0.7%)	0 (0.0%)	1.000
Heart failure (any)	241 (16.2%)	209 (15.3%)	32 (27.4%)	0.001
Hypertension	986 (66.4%)	901 (65.8%)	85 (72.6%)	0.162
Diabetes on insulin	117 (7.9%)	103 (7.5%)	14 (12.0%)	0.125
NYHA class III or IV	143 (9.6%)	122 (8.9%)	21 (17.9%)	0.003
CCS class 4 angina	7 (0.5%)	6 (0.4%)	1 (0.9%)	0.437
Myocardial infarction within 90 days	481 (32.4%)	449 (32.8%)	32 (27.4%)	0.269
Echocardiography				
LVEF (%), median [IQR]	54 [44 to 60]	54 [44 to 60]	54 [45 to 60]	0.923
LVEF 50% or less	589 (39.6%)	541 (39.5%)	48 (41.0%)	0.825
LVEF not recorded	43 (2.9%)	40 (2.9%)	3 (2.6%)	1.000
PASP over 55 mmHg	105 (7.1%)	83 (6.1%)	22 (18.8%)	<0.001
PASP not measured	896 (60.3%)	841 (61.4%)	55 (47.0%)	0.003
Predicted mortality				
EuroSCORE II (%), median [IQR]	2.0 [1.3 to 3.5]	1.9 [1.2 to 3.3]	3.9 [2.3 to 8.1]	<0.001
Additive EuroSCORE (points), median [IQR]	5 [3 to 7]	5 [3 to 7]	7 [5 to 10]	<0.001
STS PROM (%), median [IQR] (*n* = 1,076)	1.4 [0.8 to 2.6]	1.3 [0.8 to 2.4]	4.2 [2.3 to 6.8]	<0.001

Values are *n* (%) or median [IQR]; percentages are calculated within each column.

a Cockcroft-Gault estimate in patients not on dialysis.

b The registry field records shock within 24 h before surgery or on arrival in the intensive care unit after surgery.

*p* values: Mann–Whitney *U*-test for continuous variables and chi-square or Fisher exact test for categorical variables. CABG, coronary artery bypass grafting; CCS, Canadian Cardiovascular Society; IQR, interquartile range; LVEF, left ventricular ejection fraction; NYHA, New York Heart Association; PASP, pulmonary artery systolic pressure; STS PROM, Society of Thoracic Surgeons Predicted Risk of Mortality.

### 3.2 Score distributions

The median EuroSCORE II predicted mortality was 2.0% (IQR 1.3 to 3.5; mean 3.1%; range 0.5 to 52.8%). The median additive EuroSCORE was 5 points (IQR 3 to 7; mean 5.3; range 0 to 18). In the 1,076 procedures within the STS scope, the median STS PROM was 1.4% (IQR 0.8 to 2.6; mean 2.3%; range 0.2 to 42.0%). Mean predicted mortality was lower than observed mortality for all three models (7.9% in the full cohort and 6.1% in the STS subgroup).

### 3.3 Discrimination and calibration

Discrimination was acceptable for both EuroSCORE models and good for STS PROM (Table 2, Figures 1, 2). In the full cohort, the AUC was 0.736 (95% CI 0.687 to 0.785) for EuroSCORE II and 0.733 (95% CI 0.684 to 0.783) for the additive EuroSCORE (difference 0.003, *p* = 0.87). In its subgroup, STS PROM had an AUC of 0.815 (95% CI 0.757 to 0.873). In the same 1,076 procedures, STS PROM discriminated better than EuroSCORE II (AUC 0.735; difference 0.080, *p* = 0.001) and the additive EuroSCORE (AUC 0.723; difference 0.092, *p* < 0.001); this same-cohort comparison is shown in Figure 2A, and the two EuroSCORE models in the full cohort in Figure 2B.

**Table 2:** Discrimination and calibration of the three risk models.

Score	n (deaths)	Mean predicted vs observed (%)	AUC (95% CI)	O/E (95% CI)	CITL (95% CI)	Calibration slope (95% CI)	HL *χ*^2^ (p)	Brier; ICI
EuroSCORE II	1,486 (117)	3.1 vs 7.9	0.736 (0.687 to 0.785)	2.50 (2.09 to 3.00)	1.03 (0.83 to 1.23)	1.04 (0.82 to 1.26)	128.4 (<0.001)	0.070; 0.047
Additive EuroSCORE^a^	1,486 (117)	5.3 vs 7.9	0.733 (0.684 to 0.783)	1.50 (1.25 to 1.79)	0.44 (0.25 to 0.63)	1.94 (1.46 to 2.41)	51.4 (<0.001)	0.070; 0.029
STS PROM^b^	1,076 (66)	2.3 vs 6.1	0.815 (0.757 to 0.873)	2.72 (2.14 to 3.47)	1.11 (0.85 to 1.36)	1.31 (1.01 to 1.60)	102.5 (<0.001)	0.055; 0.040
EuroSCORE II, STS subgroup^c^	1,076 (66)	2.5 vs 6.1	0.735 (0.669 to 0.801)	2.45 (1.92 to 3.12)	0.97 (0.71 to 1.22)	1.20 (0.88 to 1.53)	79.6 (<0.001)	0.056; 0.037
Additive EuroSCORE, STS subgroup^c^	1,076 (66)	4.7 vs 6.1	0.723 (0.661 to 0.785)	1.30 (1.02 to 1.65)	0.28 (0.03 to 0.53)	1.87 (1.24 to 2.50)	21.5 (0.006)	0.056; 0.018

a Point total read as the predicted percentage mortality.

b Official STS calculator (2018 models) in procedures within their scope: isolated CABG, isolated AVR, isolated MVR, AVR plus CABG, MVR plus CABG, and mitral valve repair.

c EuroSCORE models evaluated in the same 1,076 procedures for direct comparison with STS PROM. CITL: ideal value 0, positive values indicate underestimation. Calibration slope: ideal value 1. AUC confidence intervals by the DeLong method. AUC, area under the receiver operating characteristic curve; AVR, aortic valve replacement; CABG, coronary artery bypass grafting; CI, confidence interval; CITL, calibration in the large; HL, Hosmer-Lemeshow test (10 groups); ICI, integrated calibration index; MVR, mitral valve replacement; O/E, observed to expected ratio; STS PROM, Society of Thoracic Surgeons Predicted Risk of Mortality.

**Figure 1:**
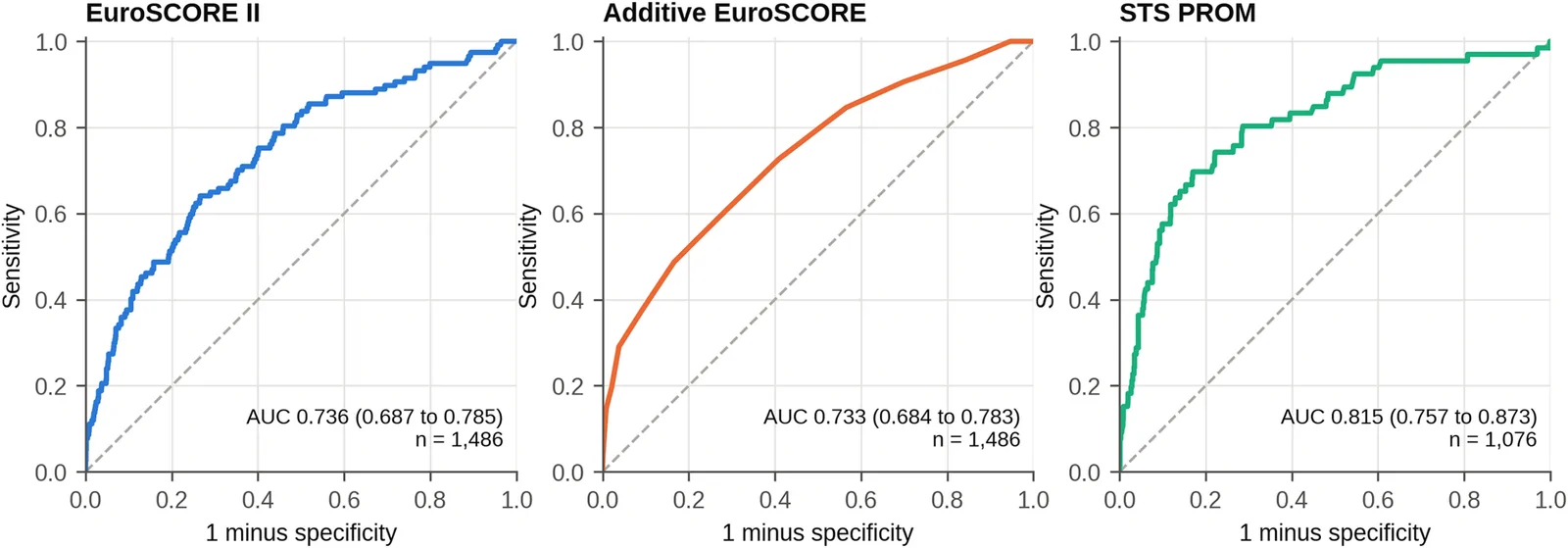
Receiver operating characteristic (ROC) curves for each risk model, with AUC and 95% deLong confidence intervals. EuroSCORE II: AUC 0.736 (0.687 to 0.785), *n* = 1,486. Additive EuroSCORE: AUC 0.733 (0.684 to 0.783), *n* = 1,486. STS PROM: AUC 0.815 (0.757 to 0.873), *n* = 1,076 (procedures within the scope of the STS 2018 models). AUC, area under the curve.

**Figure 2:**
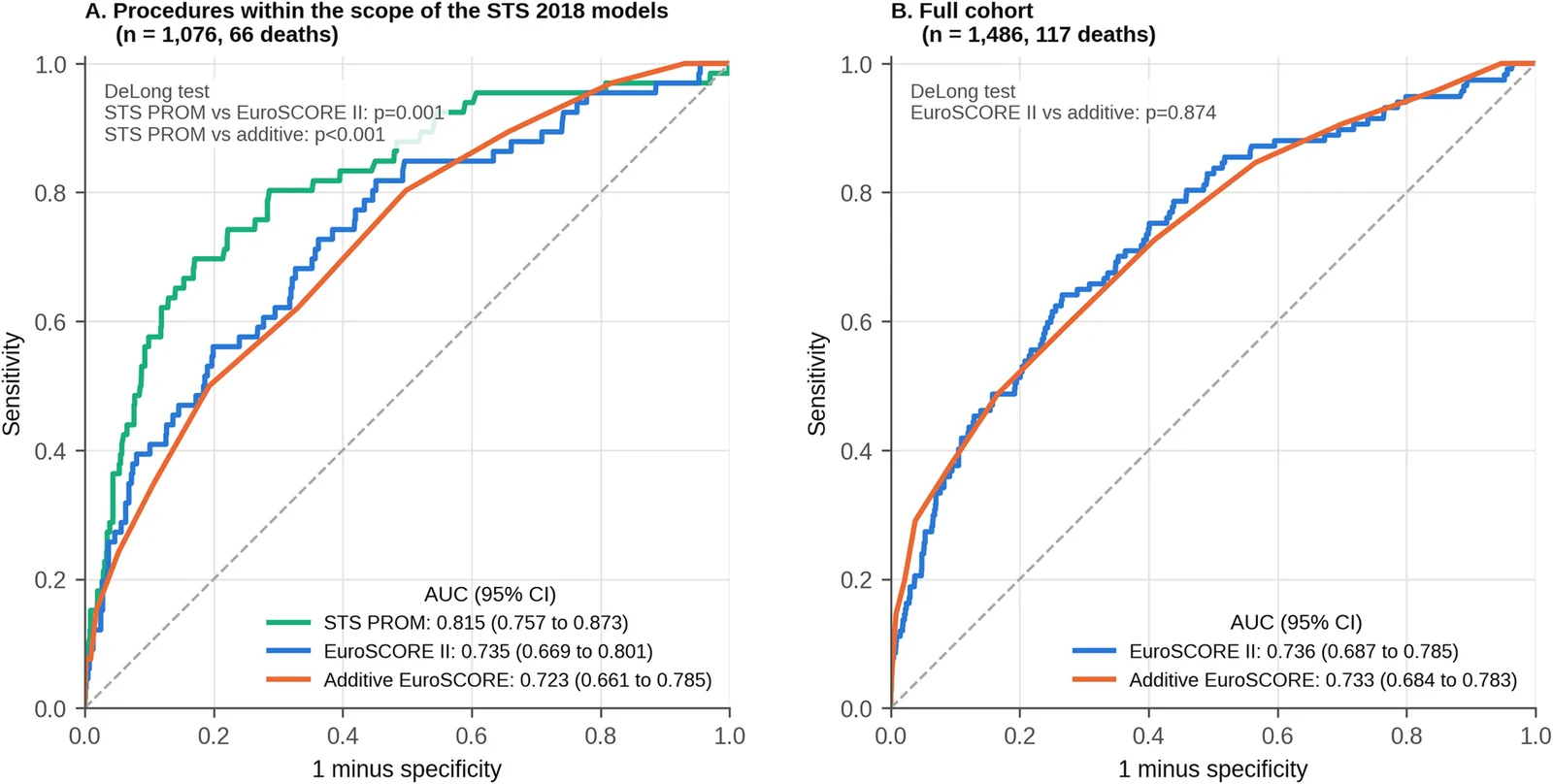
Comparative receiver operating characteristic curves. **(A)** The three models in the same patients, the 1,076 procedures within the scope of the STS 2018 models (66 deaths): STS PROM (AUC 0.815), EuroSCORE II (AUC 0.735), and the additive EuroSCORE (AUC 0.723), with the *p* values of the paired DeLong tests. **(B)** EuroSCORE II (AUC 0.736) and the additive EuroSCORE (AUC 0.733) in the full cohort (*n* = 1,486, 117 deaths). Curves are compared only within each panel. AUC, area under the curve.

All three models underestimated in-hospital mortality (Table 2, Figure 3). For EuroSCORE II, mean predicted mortality was 3.1% against an observed 7.9% (O/E 2.50 (95% CI 2.09 to 3.00); CITL 1.03 (95% CI 0.83 to 1.23)). Its calibration slope was 1.04 (95% CI 0.82 to 1.26), indicating that the spread of predicted risks was appropriate and that the miscalibration was mainly a uniform shift in baseline risk. The additive EuroSCORE predicted a mean mortality of 5.3% (O/E 1.50 (95% CI 1.25 to 1.79); CITL 0.44), with a calibration slope of 1.94 (95% CI 1.46 to 2.41), meaning that its predictions were too compressed: underestimation was small in low-risk patients and marked in high-risk patients. STS PROM predicted a mean mortality of 2.3% against an observed 6.1% in its subgroup (O/E 2.72 (95% CI 2.14 to 3.47); CITL 1.11 (95% CI 0.85 to 1.36)), with a calibration slope of 1.31 (95% CI 1.01 to 1.60). The Hosmer-Lemeshow test was significant for all three models (*p* < 0.001). Brier scores were 0.070, 0.070, and 0.055, respectively.

**Figure 3:**
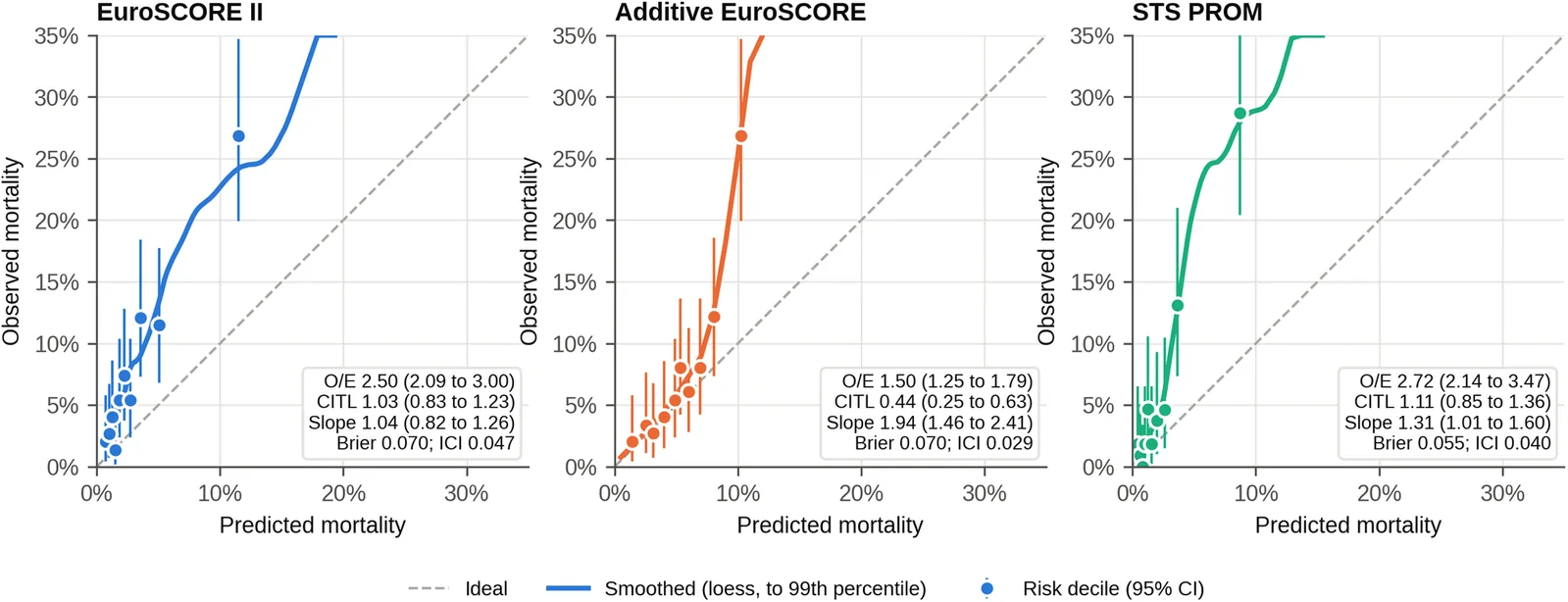
Calibration plots of observed versus predicted in-hospital mortality. Points show deciles of predicted risk with exact binomial 95% confidence intervals; the solid line is a loess smoother drawn up to the 99th percentile of predicted risk; the dashed diagonal indicates perfect calibration. For the additive EuroSCORE, the point total is read as the predicted percentage. EuroSCORE II: O/E 2.50, CITL 1.03, slope 1.04. Additive EuroSCORE: O/E 1.50, CITL 0.44, slope 1.94. STS PROM: O/E 2.72, CITL 1.11, slope 1.31. CITL, calibration in the large; ICI, integrated calibration index; O/E, observed to expected ratio.

### 3.4 Subgroup analysis

#### 3.4.1 By procedure

EuroSCORE II underestimated mortality in every procedure group (Table 3, Figure 4A), with O/E ratios ranging from 2.14 (95% CI 1.65 to 2.78) in CABG to 4.67 in thoracic aortic surgery (95% CI 2.51 to 8.68; 10 deaths) and in other procedures (95% CI 2.43 to 8.98; 9 deaths); the evidence for a difference between groups was weak (heterogeneity *p* = 0.054). The additive EuroSCORE was close to calibrated in CABG (O/E 1.28 (95% CI 0.99 to 1.67)) and aortic valve surgery (1.32 (95% CI 0.90 to 1.92)) and underestimated mortality in the remaining groups. STS PROM underestimated mortality in all STS procedure categories, with O/E ratios from 2.22 (MVR plus CABG) to 3.60 (isolated MVR) (Table 3, Figure 4B). Several groups had fewer than 15 deaths, so their estimates are imprecise.

**Table 3:** Observed to expected mortality ratios by procedure type and by STS procedure category.

Group	n	Deaths (%)	ES II pred. (%)	ES II O/E (95% CI)	ES II AUC	Additive O/E (95% CI)	STS pred. (%)	STS O/E (95% CI)
A. Procedure type (all procedures)								
CABG, isolated or combined	932	57 (6.1)	2.9	2.14 (1.65 to 2.78)	0.709	1.28 (0.99 to 1.67)	2.1	2.62 (1.97 to 3.49); *n* = 834
Aortic valve, no CABG	331	27 (8.2)	3.6	2.24 (1.54 to 3.26)	0.773	1.32 (0.90 to 1.92)	2.0	2.62 (1.41 to 4.87); *n* = 186
Mitral valve, no CABG	98	14 (14.3)	4.1	3.51 (2.08 to 5.92)	0.794	2.48 (1.47 to 4.19)	4.5	3.59 (1.87 to 6.90); *n* = 56
Thoracic aortic surgery	53	10 (18.9)	4.0	4.67 (2.51 to 8.68)	0.667	2.50 (1.35 to 4.65)	n/a	n/a
Other procedures	72	9 (12.5)	2.7	4.67 (2.43 to 8.98)	·	2.48 (1.29 to 4.77)	n/a	n/a
All procedures	**1,486**	**117** (**7.9)**	**3**.**1**	**2.50** **(2.09 to 3.00)**	**0**.**736**	**1.50** **(1.25 to 1.79)**	**2**.**3**	**2.72 (2.14 to 3.47); *n*** **=** **1,076**
B. STS procedure categories								
Isolated CABG	757	34 (4.5)	2.4	1.89 (1.35 to 2.64)	0.665	1.03 (0.73 to 1.44)	1.7	2.65 (1.89 to 3.70)
Isolated AVR	186	10 (5.4)	2.2	2.50 (1.35 to 4.65)	0.704	1.00 (0.54 to 1.85)	2.0	2.62 (1.41 to 4.87)
Isolated MVR	54	9 (16.7)	2.9	5.75 (2.99 to 11.06)		2.96 (1.54 to 5.69)	4.6	3.60 (1.87 to 6.91)
AVR plus CABG	51	6 (11.8)	4.2	2.81 (1.26 to 6.25)		1.95 (0.88 to 4.35)	3.8	3.12 (1.40 to 6.95)
MVR plus CABG	26	7 (26.9)	4.6	5.84 (2.78 to 12.25)		4.19 (2.00 to 8.79)	12.1	2.22 (1.06 to 4.65)
Mitral valve repair	2	0 (0.0)	1.0	no deaths		no deaths	0.3	no deaths

Panel A includes all 1,486 procedures; the STS columns refer to the procedures within the scope of the STS 2018 models in each group (n shown). Panel B includes the 1,076 procedures evaluated with STS PROM. AUC is shown only for groups with at least 10 deaths and 10 survivors; estimates based on fewer than 15 deaths are imprecise. Predicted values are group means. AUC, area under the receiver operating characteristic curve; AVR, aortic valve replacement; CABG, coronary artery bypass grafting; ES II, EuroSCORE II; MVR, mitral valve replacement; n/a, not applicable (outside the scope of the STS models); O/E, observed to expected ratio; pred., predicted mortality; STS, Society of Thoracic Surgeons Predicted Risk of Mortality.

Bold values indicate the results for the whole cohort.

**Figure 4:**
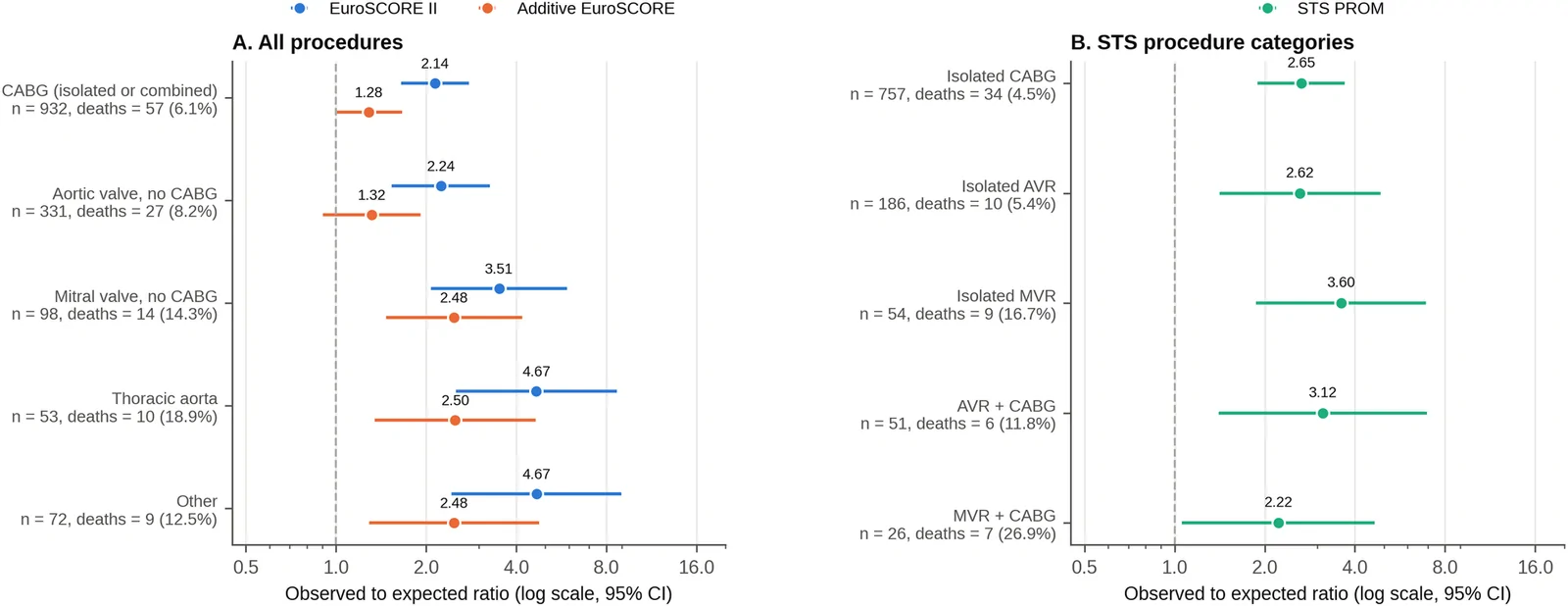
Observed to expected (O/E) mortality ratios with 95% confidence intervals (log scale). **(A)** EuroSCORE II (blue) and additive EuroSCORE (orange) by procedure type in the full cohort. **(B)** STS PROM (green) by STS procedure category; the two mitral valve repairs, without deaths, are not shown. The dashed vertical line indicates an O/E ratio of 1. Values above the markers are O/E ratios; *n* and observed in-hospital mortality are given for each group.

#### 3.4.2 EuroSCORE II risk strata

The underestimation by EuroSCORE II was similar across strata of predicted risk (Table 4): the O/E ratio was 2.48 (95% CI 1.65 to 3.73) below 2%, 2.67 (1.98 to 3.59) from 2% to 5%, 2.79 (1.96 to 3.97) from 5% to 10%, and 1.96 (1.26 to 3.03) at 10% or more, where observed mortality was 32.8% against a mean prediction of 16.8%. In the lowest stratum, observed mortality was 3.1% against a mean prediction of 1.3%. By urgency, the EuroSCORE II O/E ratio was 1.70 (95% CI 0.85 to 3.40) in elective procedures (8 deaths) and 2.57 (95% CI 2.12 to 3.11) in urgent procedures (Supplementary Table S3).

**Table 4:** Observed and predicted mortality by EuroSCORE II risk stratum.

EuroSCORE II risk stratum	*n* (% of cohort)	Deaths	Observed mortality (%)	Mean predicted mortality (%)	O/E (95% CI)
Low (below 2%)	733 (49.3%)	23	3.1	1.3	2.48 (1.65 to 3.73)
Moderate (2 to 5%)	530 (35.7%)	43	8.1	3.0	2.67 (1.98 to 3.59)
High (5 to 10%)	162 (10.9%)	31	19.1	6.9	2.79 (1.96 to 3.97)
Very high (10% or more)	61 (4.1%)	20	32.8	16.8	1.96 (1.26 to 3.03)

Strata are defined by the recalculated EuroSCORE II. O/E, observed to expected ratio; CI, confidence interval.

### 3.5 Annual trends

Annual in-hospital mortality ranged from 4.7% in 2019 to 12.6% in 2022 (Table 5, Figure 5). EuroSCORE II underestimated mortality in every calendar year from 2019 to 2025, with O/E ratios from 1.89 (2019) to 4.33 (2022), and there was no significant trend over time (rate ratio per year 1.04, *p* = 0.42). None of the 16 procedures performed in 2026 was followed by in-hospital death.

**Table 5:** Annual in-hospital mortality and predicted risk, January 2019 to February 2026.

Year	*n*	Deaths (%)	ES II pred. (%)	ES II O/E (95% CI)	Additive O/E (95% CI)	STS n (deaths)	STS pred. (%)	STS O/E (95% CI)
2019	236	11 (4.7)	2.5	1.89 (1.04 to 3.41)	0.93 (0.52 to 1.68)	187 (7)	2.0	1.91 (0.91 to 4.01)
2020	272	23 (8.5)	4.1	2.08 (1.38 to 3.13)	1.55 (1.03 to 2.33)	217 (14)	2.8	2.34 (1.39 to 3.95)
2021	227	24 (10.6)	4.4	2.41 (1.62 to 3.60)	1.74 (1.16 to 2.59)	152 (13)	3.1	2.78 (1.61 to 4.79)
2022	167	21 (12.6)	2.9	4.33 (2.82 to 6.63)	2.35 (1.53 to 3.60)	106 (10)	2.2	4.36 (2.34 to 8.10)
2023	194	15 (7.7)	2.7	2.86 (1.73 to 4.75)	1.57 (0.95 to 2.60)	150 (8)	1.8	2.92 (1.46 to 5.85)
2024	187	14 (7.5)	2.8	2.71 (1.61 to 4.58)	1.40 (0.83 to 2.37)	119 (7)	2.2	2.69 (1.28 to 5.65)
2025	187	9 (4.8)	2.3	2.11 (1.10 to 4.05)	1.07 (0.56 to 2.06)	132 (7)	1.5	3.46 (1.65 to 7.26)
2026^a^	16	0 (0.0)	2.2	no deaths	no deaths	13 (0)	2.1	no deaths
**Total**	**1,486**	**117** (**7.9)**	**3**.**1**	**2.50** **(2.09 to 3.00)**	**1.50** **(1.25 to 1.79)**	**1,076 (66)**	**2**.**3**	**2.72** **(2.14 to 3.47)**

a January and February 2026 only.

STS columns refer to the procedures within the scope of the STS 2018 models. There was no trend in the EuroSCORE II O/E ratio over time (Poisson rate ratio per year 1.04, *p* = 0.42). ES II, EuroSCORE II; O/E, observed to expected ratio; pred., mean predicted mortality; STS, Society of Thoracic Surgeons Predicted Risk of Mortality.

Bold values indicate the results for the whole cohort.

**Figure 5:**
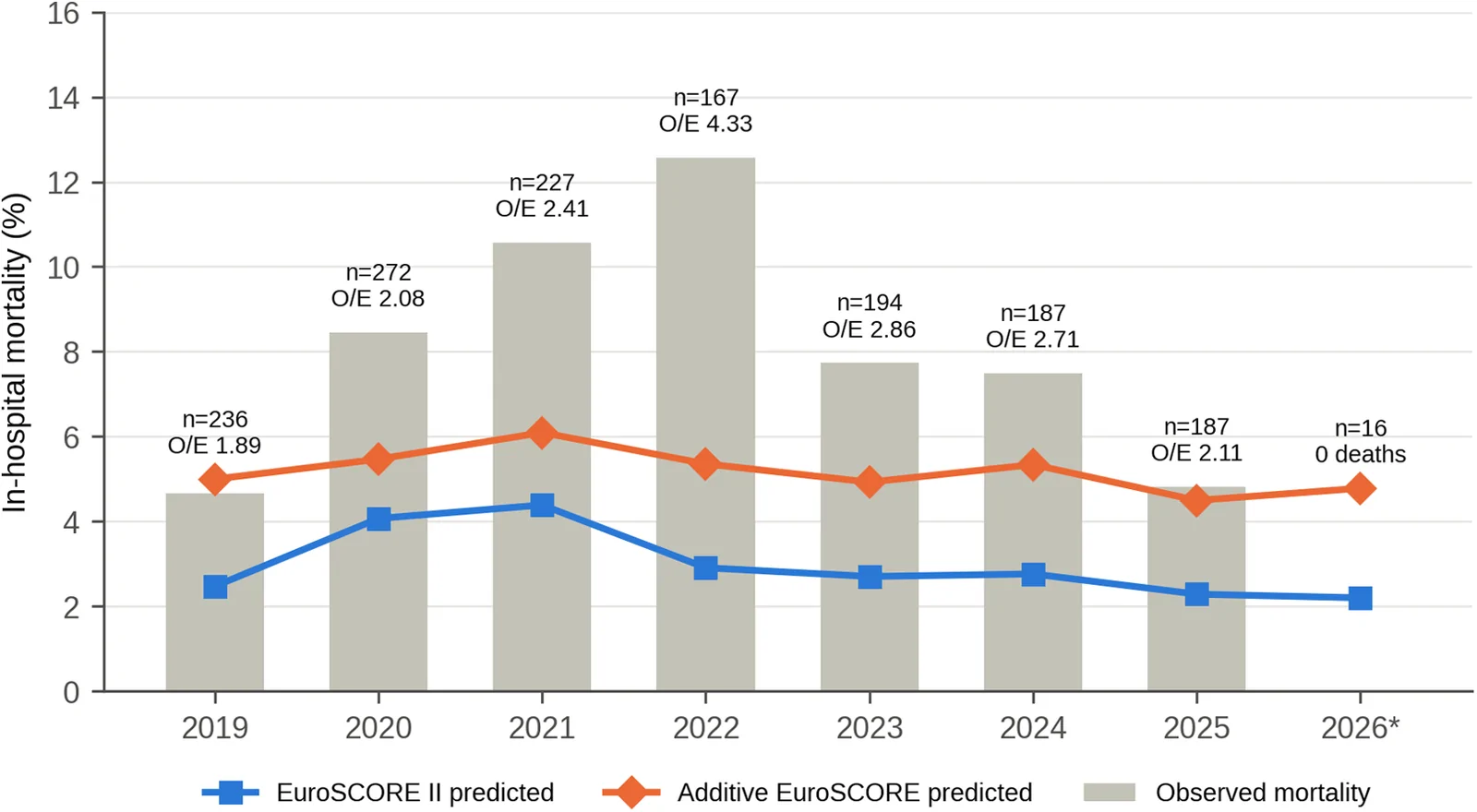
Annual in-hospital mortality (bars) and mean predicted mortality by EuroSCORE II (squares) and the additive EuroSCORE (diamonds), January 2019 to February 2026. Labels show the number of procedures and the EuroSCORE II O/E ratio for each year. *January and February 2026 only (16 procedures, no deaths).

### 3.6 Sensitivity analyses

In sensitivity analyses, the EuroSCORE II O/E ratio ranged from 2.37 (complete cases, *n* = 1,358) to 3.05 (any chronic kidney disease assigned the coefficient for creatinine clearance of 50 mL/min or less), and the AUC from 0.734 to 0.759 (Supplementary Table S4, Supplementary Figure S1). Multiple imputation of missing creatinine, weight, and ejection fraction gave an O/E ratio of 2.43 (95% CI 2.03 to 2.92). For STS PROM, excluding the 2026 procedures gave an O/E ratio of 2.75 (95% CI 2.16 to 3.50), and in isolated CABG the O/E ratio was 2.65 (95% CI 1.89 to 3.70) with an AUC of 0.749.

## 4 Discussion

To our knowledge, this is the first external validation of EuroSCORE II, the additive EuroSCORE, and STS PROM in a cardiac surgery centre located at high altitude (2,600 m asl). All three models discriminated acceptably, and STS PROM discriminated best, but all three underestimated in-hospital mortality: EuroSCORE II and STS PROM by a factor of about 2.5, and the additive EuroSCORE by a factor of 1.5. These findings are relevant to patient counselling, surgical planning, and quality benchmarking in high-altitude centres in Latin America.

The discrimination of EuroSCORE II (AUC 0.736) and the additive EuroSCORE (AUC 0.733) was somewhat lower than in their development cohorts and in most European and North American validations, whereas that of STS PROM (AUC 0.815) was similar to its development data (4, 5, 7, 26–29). The ranking of patients by risk was therefore largely preserved despite differences in population and setting, in line with other external validation studies (8, 9, 13, 14, 26, 27, 30–32). The better discrimination of STS PROM in the same patients probably reflects its larger set of predictors, which includes laboratory values, coronary anatomy, and cardiac presentation, and its procedure-specific equations, although it applies only to a subset of operations. The additive EuroSCORE discriminated as well as EuroSCORE II in our cohort, consistent with reports that both models rank patients similarly (1, 26).

Calibration, in contrast, was poor. The underestimation by EuroSCORE II and STS PROM was about 2.5-fold and was consistent across procedure types, risk strata, and calendar years. Because the calibration slope of EuroSCORE II was close to 1, the error lies mainly in the baseline risk, which is what a model developed elsewhere would miss if local mortality is higher for reasons its predictors do not capture. Several explanations are possible, and our design cannot distinguish between them. Chronic hypoxia at 2,600 m asl is associated with pulmonary vasoconstriction, a higher right ventricular afterload, and haematological changes that could increase perioperative risk independently of the variables included in these scores (15). However, the excess mortality may equally reflect differences in case mix that the scores capture incompletely, the high proportion of urgent procedures (81%), delays in access to surgery, and local perioperative practice and resources. Although the registry did not record each patient's place of residence, at least 97% of the patients treated in our hospital live in Bogotá or in nearby towns, and only a small proportion are not acclimatised to high altitude. Our results therefore show that the models are miscalibrated in this centre; the excess mortality may reflect patient complexity, available resources, and the small proportion of patients admitted electively from the outpatient setting, and whether altitude itself contributes to it cannot be established with this design. Of note, lower preoperative haematocrit and haemoglobin, rather than erythrocytosis, added prognostic information to EuroSCORE II (Supplementary Material); anaemia, which is underestimated at altitude when sea-level thresholds are used, deserves attention in future models.

The additive EuroSCORE is usually reported to overestimate risk in contemporary cohorts because of improvements in anaesthesia, perfusion, and surgical technique since the 1990s (13, 26). In our cohort it underestimated mortality overall (O/E 1.50), with near-adequate calibration in CABG and aortic valve surgery and a calibration slope of 1.94, which shows that its point system does not assign enough risk to the sickest patients. In a centre with higher mortality than the European development cohort, the historical overestimation of the additive score and the local excess risk may partly cancel out, and this should not be mistaken for good performance.

Previous Latin American validation studies, conducted mainly in Argentina (31, 33), Brazil (33), and Mexico (30), reported EuroSCORE II AUC values between 0.76 and 0.81 and O/E ratios between 1.0 and 2.46, which are close to ours for discrimination but in most cases indicate smaller underestimation. These cohorts include little representation from the Andean region.

In Colombia, previous studies of EuroSCORE II have yielded conflicting results: some reported performance comparable to international benchmarks (10, 34), whereas a study of CABG reported an AUC of 0.55 (95% CI 0.48 to 0.63) and an O/E ratio of 4.2 (12). Our CABG group also showed underestimation (O/E 2.14), with better discrimination (AUC 0.709). These differences may reflect the limitations of retrospective single-centre data and differences in how the score was implemented. Evidence on the effect of high altitude remains scarce: studies of high-altitude populations in Tibet did not directly assess these models, and one even suggested that high-altitude adaptation might be protective (15, 16), which contrasts with our observations.

This study has several strengths: a large consecutive cohort from a prospective registry, recalculation of EuroSCORE II and the additive EuroSCORE with the published equations and all their items, STS PROM values obtained from the official calculator, a complete set of calibration measures, and sensitivity analyses showing that the underestimation does not depend on how renal function, missing data, or registry definitions were handled (EuroSCORE II O/E 2.37 to 3.05). It also has limitations. First, it is a single-centre study, and its results may not apply to other high-altitude centres, such as those in Quito (2,850 m) or La Paz (3,600 m). Second, some registry fields do not match the score definitions exactly: few patients were flagged with extracardiac arteriopathy or poor mobility, cardiogenic shock also includes shock on arrival in the intensive care unit, and urgency reflects in-hospital status before surgery. Third, several STS inputs were unavailable or approximated (for example, non-insulin-treated diabetes, timing of antiplatelet therapy, and payer), which may have lowered the predicted risk. Fourth, the outcome was in-hospital rather than STS operative mortality; including deaths after discharge would, if anything, increase the STS underestimation. Finally, the calibration slope was estimated with limited precision, and several subgroups had few deaths, so their estimates should be interpreted with caution.

Our findings indicate that EuroSCORE II and STS PROM should not be used at face value for counselling or benchmarking in this high-altitude centre without local recalibration. In a temporal validation, updating only the EuroSCORE II intercept with 2019 to 2021 data reduced the O/E ratio in 2022 to 2026 from 2.97 to 1.33 without loss of discrimination, and re-estimating all coefficients added little (AUC 0.736 vs. 0.727; Supplementary Material). A recalibrated EuroSCORE II is therefore a practical interim tool, whereas identifying altitude-related predictors will require a model developed and externally validated in several high-altitude centres with a larger number of events.

## 5 Conclusions

EuroSCORE II, the additive EuroSCORE, and STS PROM showed acceptable to good discrimination but underestimated in-hospital mortality in a Colombian cardiac surgery centre located at 2,600 m asl, EuroSCORE II and STS PROM by about 2.5-fold and the additive EuroSCORE by about 1.5-fold. This study cannot determine whether the excess mortality reflects altitude, case mix, or local care. Local recalibration should precede the use of these models for counselling and benchmarking in this setting, and multicentre studies in high-altitude centres are needed to develop and validate specific models.

## Data Availability

The datasets presented in this study can be found in online repositories. The names of the repository/repositories and accession number(s) can be found below: https://doi.org/10.5281/zenodo.22797059.

## References

[1] Zingone B Pappalardo A Dreas L. Logistic versus additive EuroSCORE. A comparative assessment of the two models in an independent population sample. Eur J Cardiothorac Surg. (2004) 26:1134–40. 10.1016/j.ejcts.2004.09.003 15541974

[2] Saia F Lauck S Durand E Muir DF Spence M Vasa-Nicotera M. The implementation of a streamlined TAVI patient pathway across five European countries: BENCHMARK registry. Clin Res Cardiol. (2026) 115:1107–19. 10.1007/s00392-025-02638-z 40261426 PMC13249708

[3] Nashef SA Roques F Michel P Gauducheau E Lemeshow S Salamon R. European system for cardiac operative risk evaluation (EuroSCORE). Eur J Cardiothorac Surg. (1999) 16:9–13. 10.1016/s1010-7940(99)00134-7 10456395

[4] Nashef SAM Roques F Sharples LD Nilsson J Smith C Goldstone AR. EuroSCORE II. Eur J Cardiothorac Surg. (2012) 41:734–45. 10.1093/ejcts/ezs043 22378855

[5] O’Brien SM Feng L He X Xian Y Jacobs JP Badhwar V. The society of thoracic surgeons 2018 adult cardiac surgery risk models: part 2—statistical methods and results. Ann Thorac Surg. (2018) 105:1419–28. 10.1016/j.athoracsur.2018.03.003 29577924

[6] O'Brien SM Shahian DM Filardo G Ferraris VA Haan CK Rich JB. The society of thoracic surgeons 2008 cardiac surgery risk models: part 2—isolated valve surgery. Ann Thorac Surg. (2009) 88:S23–42. 10.1016/j.athoracsur.2009.05.056 19559823

[7] Shahian DM Jacobs JP Badhwar V Kurlansky PA Furnary AP Cleveland JC. The society of thoracic surgeons 2018 adult cardiac surgery risk models: part 1—background, design considerations, and model development. Ann Thorac Surg. (2018) 105:1411–8. 10.1016/j.athoracsur.2018.03.002 29577925

[8] Wessler BS Nelson J Park JG McGinnes H Gulati G Brazil R. External validations of cardiovascular clinical prediction models: a large-scale review of the literature. Circ Cardiovasc Qual Outcomes. (2021) 14:e007858. 10.1161/CIRCOUTCOMES.121.007858 34340529 PMC8366535

[9] Wang C Li X Lu F- Xu J- Tang H Han L. Comparison of six risk scores for in-hospital mortality in Chinese patients undergoing heart valve surgery. Heart Lung Circ. (2013) 22:612–7. 10.1016/j.hlc.2013.03.084 23619196

[10] Jaramillo JM. Capacidad predictiva del EuroSCORE II en una cohorte de pacientes de cirugía cardiovascular en una institución de cuarto nivel de Bogotá. Bogotá: Universidad del Rosario (2015).

[11] Garzón-Rodríguez JD Torres-Gómez PL Macias KA Suárez-Tequia L Noriega-Acosta F Rodríguez-Aparicio EE. Cardiac surgery in older adults: a retrospective cohort study of outcomes and risk score performance in octogenarians versus patients aged 65–79 years. BMC Geriatr. (2025) 25:788. 10.1186/s12877-025-06416-4 PMC12533456 41107805

[12] Rodríguez Lima DR Rodríguez Aparicio EE Otálora González L Hernández DC González-Muñoz A. Performance of the EuroSCORE in coronary artery bypass graft in Colombia, a middle-income country: a retrospective cohort. World J Cardiol. (2025) 17:100506. 10.4330/wjc.v17.i3.100506 PMC11947952 40161567

[13] Hsieh CH Peng SK Tsai TC Shih YR Peng SY. Prediction for major adverse outcomes in cardiac surgery: comparison of three prediction models. J Formos Med Assoc. (2007) 106:759–67. 10.1016/S0929-6646(08)60037-6 17908665

[14] Zhang G Wang C Wang L Lu F Li B Han L. Validation of EuroSCORE II in Chinese patients undergoing heart valve surgery. Heart Lung Circ. (2013) 22:606–11. 10.1016/j.hlc.2012.12.012 23375874

[15] Zhu Q Duan H Liu Z Li Y Zhang Y Labaciren L. The incidence and risk factors of perioperative cardiac complications in noncardiac major surgery in high-altitude areas: a prospective trial in Tibet autonomous region, China. Front Cardiovasc Med. (2023) 10:1158711. 10.3389/fcvm.2023.1158711 37077733 PMC10106712

[16] Lei L Liu M Ma D Lei X Zeng S Li P. Cardioprotective effects of high-altitude adaptation in cardiac surgical patients: a retrospective cohort study with propensity score matching. Front Cardiovasc Med. (2024) 11:1347552. 10.3389/fcvm.2024.1347552 38628317 PMC11019029

[17] Collins GS Reitsma JB Altman DG Moons KGM. Transparent reporting of a multivariable prediction model for individual prognosis or diagnosis (TRIPOD): the TRIPOD statement. Br Med J. (2015) 350:g7594. 10.1136/bmj.g7594 25569120

[18] Jacobs JP Shahian DM Badhwar V Thibault DP Thourani VH Rankin JS. The society of thoracic surgeons 2021 adult cardiac surgery risk models for multiple valve operations. Ann Thorac Surg. (2022) 113:511–8. 10.1016/j.athoracsur.2021.03.089 33844993

[19] Cockcroft DW Gault MH. Prediction of creatinine clearance from serum creatinine. Nephron. (2008) 16:31–41. 10.1159/000180580 1244564

[20] Semenkovich N. sts-risk-calculator-cli: command line interface to the STS short-term risk calculator. GitHub (2022). Available online at: https://github.com/semenko/sts-risk-calculator-cli. (Accessed 15 September, 2026)

[21] DeLong ER DeLong DM Clarke-Pearson DL. Comparing the areas under two or more correlated receiver operating characteristic curves: a nonparametric approach. Biometrics. (1988) 44:837–45. 10.2307/2531595 3203132

[22] Van Calster B McLernon DJ Van Smeden M Wynants L Steyerberg EW. Calibration: the achilles heel of predictive analytics. BMC Med. (2019) 17:230. 10.1186/s12916-019-1466-7 31842878 PMC6912996

[23] Austin PC Steyerberg EW. The integrated calibration index (ICI) and related metrics for quantifying the calibration of logistic regression prediction models. Stat Med. (2019) 38:4051–65. 10.1002/sim.8281 31270850 PMC6771733

[24] Riley RD Debray TPA Collins GS Archer L Ensor J Van Smeden M. Minimum sample size for external validation of a clinical prediction model with a binary outcome. Stat Med. (2021) 40:4230–47. 10.1002/sim.9025 34031906

[25] Janssen KJM Moons KGM Kalkman CJ Grobbee DE Vergouwe Y. Updating methods improved the performance of a clinical prediction model in new patients. J Clin Epidemiol. (2008) 61:76–86. 10.1016/j.jclinepi.2007.04.018 18083464

[26] Garcia-Valentin A Mestres CA Bernabeu E Bahamonde JA Martín I Rueda C. Validation and quality measurements for EuroSCORE and EuroSCORE II in the Spanish cardiac surgical population: a prospective, multicentre study. Eur J Cardiothorac Surg. (2016) 49:399–405. 10.1093/ejcts/ezv090 25762397

[27] Hassan J Usman N Salman M Ali A Shaukat M Khan H. Assessing the validity of society of thoracic surgeons (STS) score in predicting stroke risk among patients undergoing cardiothoracic surgery at a tertiary hospital in Pakistan: a retrospective cohort study. J Cardiothorac Surg. (2025) 20:187. 10.1186/s13019-025-03350-9 40217312 PMC11987283

[28] Moscarelli M Bianchi G Margaryan R Cerillo A Farneti P Murzi M. Accuracy of EuroSCORE II in patients undergoing minimally invasive mitral valve surgery. Interact Cardiovasc Thorac Surg. (2015) 21:748–53. 10.1093/icvts/ivv265 26403175

[29] D Dedda U Pelissero G Agnelli B D Vincentiis C Castelvecchio S Ranucci M. Accuracy, calibration and clinical performance of the new EuroSCORE II risk stratification system. Eur J Cardiothorac Surg. (2013) 43:27–32. 10.1093/ejcts/ezs196 22822108

[30] Rodríguez-Chávez LL Figueroa-Solano J Muñoz-Consuegra CE Avila-Vanzzini N Kuri-Alfaro J. EuroSCORE subestima el riesgo de mortalidad en cirugía cardiaca valvular de población mexicana. Arch Cardiol México. (2017) 87:18–25. 10.1016/j.acmx.2016.07.001 27495386

[31] Borracci RA Rubio M Celano L Ingino CA Allende NG Ahuad Guerrero RA. Prospective validation of EuroSCORE II in patients undergoing cardiac surgery in argentinean centres. Interact Cardiovasc Thorac Surg. (2014) 18:539–43. 10.1093/icvts/ivt550 24491683

[32] Gómez de Antonio D Crowley Carrasco S Romero Román A Royuela A Gil Barturen M Obiols C. External validation of the European society of thoracic surgeons morbidity and mortality risk models. Eur J Cardiothorac Surg. (2022) 62:ezac170. 10.1093/ejcts/ezac170 35301527

[33] Cerda-Núnez C Yánez-Lillo J Seguel E Guinez-Molinos S. Performance of EuroSCORE II in Latin America: a systematic review. Rev Med Chil. (2022) 150:424–30. 10.4067/S0034-98872022000400424 36155751

[34] Molina RS Molina-Rodríguez MA Rincón FM Maldonado JD. Cardiac operative risk in Latin America: a comparison of machine learning models vs EuroSCORE-II. Ann Thorac Surg. (2022) 113:92–9. 10.1016/j.athoracsur.2021.02.052 33689741

