# Supplementary material for: External validation of EuroSCORE II, additive EuroSCORE, and the STS predicted risk of mortality at 2,600 meters above sea level: a retrospective cohort study from a Latin American cardiac surgery centre
Source: Front Cardiovasc Med. 2026 Oct 7;13:1840998. doi: 10.3389/fcvm.2026.1840998 (PMC13683153; doi:10.3389/fcvm.2026.1840998)
Supplement: Supplementary file 1 [file Datasheet1.docx]

**Supplementary Material**

**External Validation of EuroSCORE II, Additive EuroSCORE, and the STS Predicted Risk of Mortality at 2,600 Meters Above Sea Level: A Retrospective Cohort Study from a Latin American Cardiac Surgery Centre**

*Rodríguez-Lima DR, Garzón-Rodríguez JD, Torres-Gómez PL, Rubio-Ramos C, Gómez-Cortes LA, Campo-Bautista EM, Rodriguez-Aparicio EE, Toro-Egas JD*

**Contents**

S1. Mapping of EuroSCORE II and additive EuroSCORE items to registry fields (Table S1)

S2. Mapping of registry fields to the STS calculator inputs (Table S2)

S3. Additional subgroup analyses (Table S3)

S4. Sensitivity analyses (Table S4, Figure S1)

S5. Procedures outside the scope of the STS 2018 models (Table S5)

S6. Precision, temporal validation and updating of EuroSCORE II, and altitude-related markers (Tables S6 to S10, Figure S2)

# S1. Mapping of EuroSCORE II and additive EuroSCORE items to registry fields

EuroSCORE II was calculated with the published logistic equation (Nashef et al., 2012): predicted mortality = e^y / (1 + e^y), where y = −5.324537 + Σ βi Xi. All 18 risk factors were included. Table S1 lists, for each item, the registry field and rule used and the number of patients with the factor in the analytic cohort (N=1,486).

**Table S1.** EuroSCORE II items, coefficients, and registry mapping

| **EuroSCORE II item** | **Coefficient** | **Registry field and rule** | **n (%)** |
| --- | --- | --- | --- |
| Age | 0.0285181 per year; X = 1 if 60 years or younger, otherwise age minus 59 | edad (age at surgery) | median 66 years |
| Female sex | 0.2196434 | sexo = 2 | 445 (29.9%) |
| Renal impairment: on dialysis | 0.6421508 | es2_dialisis = 2 (dialysis), regardless of creatinine | 49 (3.3%) |
| Renal impairment: creatinine clearance 50 mL/min or less | 0.8592256 | Cockcroft-Gault from creatinina_preop, edad, peso_kg and sex; not on dialysis | 193 (13.0%) |
| Renal impairment: creatinine clearance 51 to 85 mL/min | 0.303553 | As above | 736 (49.5%) |
| Extracardiac arteriopathy | 0.5360268 | EuroSCORE II checkbox (aortic or carotid disease) or arteriopatia_periferica (STS form) | 59 (4.0%) |
| Poor mobility | 0.2407181 | es2_deficit_motor | 10 (0.7%) |
| Previous cardiac surgery | 1.118599 | es2_cx_previa_ninguna = 0 (any previous CABG, valve, aortic, congenital or other cardiac surgery) | 37 (2.5%) |
| Chronic lung disease | 0.1886564 | epoc (epoc_grado 1 to 4) | 67 (4.5%) |
| Active endocarditis | 0.6194522 | es2_endocarditis_activa | 44 (3.0%) |
| Critical preoperative state | 1.086517 | any es2_critico component (ventricular arrhythmia, preoperative ventilation, resuscitation, inotropes or balloon pump or assist device, acute renal failure) | 47 (3.2%) |
| Diabetes on insulin | 0.3542749 | es2_dm_insulina | 117 (7.9%) |
| NYHA class II / III / IV | 0.1070545 / 0.2958358 / 0.5597929 | es2_nyha_clase (4 missing values set to class I) | 813 (54.7%) / 127 (8.5%) / 16 (1.1%) |
| CCS class 4 angina | 0.2226147 | es2_angina_ccs4 | 7 (0.5%) |
| LV function moderate / poor / very poor | 0.3150652 / 0.8084096 / 0.9346919 | es2_fevi_preop_pct: 31 to 50% / 21 to 30% / 20% or less (43 missing set to good) | 484 (32.6%) / 91 (6.1%) / 14 (0.9%) |
| Recent myocardial infarction (90 days or less) | 0.1528943 | iam_90d (iam_previo_tiempo codes 1 to 5) | 481 (32.4%) |
| Pulmonary hypertension moderate / severe | 0.1788899 / 0.3491475 | papse_valor_cat (echocardiographic PASP): 31 to 55 mmHg / over 55 mmHg; not measured set to absent | 342 (23.0%) / 105 (7.1%) |
| Urgency: urgent / emergency / salvage | 0.3174673 / 0.7039121 / 1.362947 | es2_urgencia recoded (registry code 1 urgent, 2 elective, 3 emergency, 4 salvage) | 1,204 (81.0%) / 18 (1.2%) / 1 (0.1%) |
| Weight of intervention: single non-CABG / two / three or more | 0.0062118 / 0.5521478 / 0.9724533 | es2_peso_procedimiento codes 2 / 3 / 4 (code 1 isolated CABG, reference) | 410 (27.6%) / 234 (15.7%) / 55 (3.7%) |
| Surgery on thoracic aorta | 0.6527205 | es2_cx_aorta_toracica | 165 (11.1%) |

Constant −5.324537. CABG, coronary artery bypass grafting; CCS, Canadian Cardiovascular Society; LV, left ventricular; NYHA, New York Heart Association; PASP, pulmonary artery systolic pressure.

The additive EuroSCORE (Nashef et al., 1999) was calculated with the original point system: age, 1 point per 5 years or part thereof from 60 years; female sex, 1; chronic pulmonary disease, 1; extracardiac arteriopathy, 2; neurological dysfunction (poor mobility), 2; previous cardiac surgery, 3; serum creatinine over 200 µmol/L (over 2.26 mg/dL) or dialysis, 2; active endocarditis, 3; critical preoperative state, 3; unstable angina (sindrome_coronario_agudo = unstable angina), 2; LVEF 30% to 50%, 1; LVEF below 30%, 3; recent myocardial infarction, 2; pulmonary artery systolic pressure over 60 mmHg, 2; emergency or salvage operation, 2; other than isolated CABG, 2; surgery on the thoracic aorta, 3; postinfarct septal rupture, 4 (not recorded). The point total was read as the predicted percentage mortality. Urgent (non-emergency) operations do not score in this model. The median score was 5 points (IQR 3 to 7).

# S2. Mapping of registry fields to the STS calculator inputs

STS PROM was obtained from the official STS Adult Cardiac Surgery Database Operative Risk Calculator (https://acsdriskcalc.research.sts.org), which implements the STS 2018 risk models, using an adapted version of an open-source batch client (github.com/semenko/sts-risk-calculator-cli). The calculator was queried in September 2026 for the 1,076 eligible procedures. No names, dates, or registry identifiers were transmitted. The option strings were checked against the live calculator before the run.

**Table S2.** Mapping of registry fields to the STS calculator inputs

| **STS calculator input** | **Registry field** | **Rule or assumption** |
| --- | --- | --- |
| Procedure | proc_* checkboxes, es2_peso_procedimiento, es2_cx_aorta_toracica | Exactly one STS procedure flag, no concomitant non-STS procedure, no thoracic aortic surgery, and a consistent EuroSCORE II weight of intervention (see Table S5) |
| Age, sex, height, weight, BMI | edad, sexo, talla_cm, peso_kg | BMI calculated from height and weight |
| Race and ethnicity | not recorded individually | Hispanic for all patients |
| Payer | not applicable | Non-U.S. plan for all patients |
| Haematocrit, white cell count, platelets | hto_preop, leucocitos_preop, plaquetas_preop | White cell count divided by 1,000 |
| Creatinine, dialysis | creatinina_preop, es2_dialisis | Dialysis if es2_dialisis = 2 |
| Ejection fraction | es2_fevi_preop_pct | Left blank if missing |
| Status | es2_urgencia | Elective, urgent, emergent, emergent salvage |
| Cardiovascular surgery incidence | evento_qx_cv_previo | Code 2 first reoperation, code 3 second reoperation |
| Previous cardiac interventions | es2_cx_previa_*, antec_stent | CABG, valve, other; previous PCI entered as not during this episode of care |
| Diabetes | es2_dm_insulina | Insulin-treated diabetes; diabetes treated otherwise is not recorded in the registry and was entered as no diabetes |
| Endocarditis | es2_endocarditis_activa | Active endocarditis |
| Chronic lung disease | epoc_grado | Codes 1 to 3 mild, moderate, severe; code 4 severity unknown |
| Cerebrovascular disease | acv_reciente_90d, acv_mayor_90d, enfermedad_carotidea | Stroke within 90 days entered as stroke within 30 days; older stroke as stroke over 30 days; carotid disease as other cerebrovascular disease; carotid stenosis right if code 1 or 2 and left if code 2 |
| Heart failure, NYHA class | falla_cardiaca_tipo, es2_nyha_clase | Acute, chronic, or both |
| Cardiac presentation | sindrome_coronario_agudo | Stable angina, unstable angina, non-ST elevation MI, STEMI; no symptoms, anginal equivalent, or other entered as none or other |
| Myocardial infarction timing | iam_previo_tiempo | 6 h or less; 6 to 24 h; 1 to 7 days; 8 to 21 days; over 21 days (registry band 22 to 90 days) |
| Coronary anatomy | enfermedad_coronaria, tronco_arterial_izq, da_izq_proximal | Number of diseased vessels 0 to 3; left main stenosis 50% or more if code 2 or higher; proximal LAD stenosis 70% or more if code 3 or higher |
| Valve disease | eco_estenosis_ao, eco_estenosis_mitral, eco_insuf_ao, eco_insuf_mitral, eco_insuf_tricusp_grado | Stenosis present or absent; regurgitation codes 0 to 4 as none, trace, mild, moderate, severe; missing as unknown |
| Arrhythmia | arritmia_fa_flutter, fa_clasificacion, arritmia_flutter, arritmia_tv, arritmia_fv, arritmia_nodo_sinusal | Arrhythmias present at admission entered as recent; atrial fibrillation code 1 paroxysmal, code 2 persistent |
| Mechanical support, shock, resuscitation | baloncontrapulsacion_preop, shock_cardiogenico, reanimacion_preop | Preoperative balloon pump (code 1); cardiogenic shock as recorded (includes shock on arrival in the intensive care unit); resuscitation within 1 hour (code 1) |
| Medications | inotropico_preop, esteroide_preop | Code 3 entered as yes; ADP inhibitors, ACE inhibitors or ARB, and glycoprotein IIb/IIIa inhibitors not available and entered as no |
| Other comorbidities | hipertension, inmunosupresion, arteriopatia_periferica, antec_radioterapia, antec_cancer, antec_familiar_cv, sahos, antec_hepatopatia, deficit_conciencia, sincope, antec_neumonia, oxigenoterapia_cronica, spa, consumo_alcohol, tabaquismo | As recorded; recent pneumonia if code 1; alcohol codes 0 to 3 as none, 1 or fewer, 2 to 7, 8 or more drinks per week; smoking code 1 current, 3 former, 2 unknown, 0 never |
| Not entered | not recorded | Concomitant tricuspid repair, aortic root abscess, prior carotid surgery, TIA, heart block |

ACE, angiotensin converting enzyme; ARB, angiotensin receptor blocker; BMI, body mass index; LAD, left anterior descending artery; MI, myocardial infarction; NYHA, New York Heart Association; PCI, percutaneous coronary intervention; STEMI, ST elevation myocardial infarction; TIA, transient ischaemic attack. Missing or approximated inputs (for example non-insulin-treated diabetes) would tend to lower predicted risk.

# S3. Additional subgroup analyses

**Table S3.** Observed to expected mortality by EuroSCORE II weight of intervention and by urgency

| **Group** | **n** | **Deaths (%)** | **ES II pred. (%)** | **ES II O/E (95% CI)** | **ES II AUC (95% CI)** | **Additive O/E (95% CI)** | **STS O/E (95% CI)** |
| --- | --- | --- | --- | --- | --- | --- | --- |
| **EuroSCORE II weight of intervention** |  |  |  |  |  |  |  |
| Isolated CABG | 787 | 38 (4.8) | 2.4 | 2.04 (1.48 to 2.80) | 0.673 (0.58 to 0.76) | 1.11 (0.80 to 1.52) | 2.65 (1.89 to 3.70); n=757 |
| Single non-CABG procedure | 410 | 36 (8.8) | 2.4 | 3.59 (2.59 to 4.97) | 0.750 (0.66 to 0.84) | 1.53 (1.10 to 2.12) | 3.01 (1.92 to 4.71); n=242 |
| Two procedures | 234 | 33 (14.1) | 5.4 | 2.61 (1.85 to 3.67) | 0.713 (0.62 to 0.81) | 2.07 (1.47 to 2.91) | 2.56 (1.49 to 4.41); n=77 |
| Three or more procedures | 55 | 10 (18.2) | 9.8 | 1.86 (1.00 to 3.45) | 0.748 (0.56 to 0.93) | 2.38 (1.28 to 4.41) | n/a |
| **Urgency** |  |  |  |  |  |  |  |
| Elective | 263 | 8 (3.0) | 1.8 | 1.70 (0.85 to 3.40) | not estimated | 0.66 (0.33 to 1.33) | 2.13 (0.89 to 5.11); n=164 |
| Urgent | 1,204 | 103 (8.6) | 3.3 | 2.57 (2.12 to 3.11) | 0.729 (0.68 to 0.78) | 1.60 (1.32 to 1.95) | 2.78 (2.16 to 3.59); n=909 |
| Emergency | 18 | 6 (33.3) | 9.8 | 3.40 (1.53 to 7.57) | not estimated | 3.33 (1.50 to 7.42) | 6.30 (0.89 to 44.73); n=2 |
| Salvage | 1 | 0 (0.0) | 14.2 | no deaths | not estimated | no deaths | no deaths (n=1) |

AUC is shown only for groups with at least 10 deaths and 10 survivors. AUC, area under the receiver operating characteristic curve; ES II, EuroSCORE II; n/a, not applicable; O/E, observed to expected ratio; pred., mean predicted mortality; STS, STS PROM in the procedures within the scope of the STS 2018 models.

# S4. Sensitivity analyses

**Table S4.** Sensitivity analyses

| **Scenario** | **n** | **Deaths** | **AUC (95% CI)** | **O/E (95% CI)** | **CITL** | **Slope** |
| --- | --- | --- | --- | --- | --- | --- |
| **EuroSCORE II** |  |  |  |  |  |  |
| **Primary analysis** | **1,486** | **117** | **0.736 (0.687 to 0.785)** | **2.50 (2.09 to 3.00)** | **1.03** | **1.04** |
| Renal: any chronic kidney disease assigned the coefficient for creatinine clearance 50 mL/min or less | 1,486 | 117 | 0.759 (0.710 to 0.808) | 3.05 (2.55 to 3.66) | 1.25 | 1.15 |
| Renal: creatinine clearance category recorded in the registry | 1,486 | 117 | 0.736 (0.686 to 0.785) | 2.51 (2.09 to 3.01) | 1.03 | 1.05 |
| Missing creatinine clearance set to 51 to 85 mL/min | 1,486 | 117 | 0.742 (0.694 to 0.791) | 2.47 (2.06 to 2.96) | 1.02 | 1.07 |
| Missing creatinine clearance set to 50 mL/min or less | 1,486 | 117 | 0.748 (0.700 to 0.796) | 2.39 (1.99 to 2.86) | 0.98 | 1.10 |
| Missing LVEF assigned to moderate (31 to 50%) | 1,486 | 117 | 0.736 (0.686 to 0.785) | 2.49 (2.07 to 2.98) | 1.02 | 1.05 |
| Multiple imputation of creatinine, weight and LVEF (average of 20 imputations) | 1,486 | 117 | 0.746 (0.697 to 0.794) | 2.43 (2.03 to 2.92) | 1.00 | 1.08 |
| Complete cases only (creatinine, weight and LVEF recorded) | 1,358 | 104 | 0.742 (0.690 to 0.793) | 2.37 (1.96 to 2.88) | 0.97 | 1.08 |
| Extracardiac arteriopathy: EuroSCORE II form field only | 1,486 | 117 | 0.740 (0.691 to 0.789) | 2.57 (2.15 to 3.08) | 1.06 | 1.08 |
| Poor mobility: also any previous stroke | 1,486 | 117 | 0.735 (0.686 to 0.784) | 2.46 (2.05 to 2.95) | 1.01 | 1.03 |
| Critical state: also preoperative balloon pump | 1,486 | 117 | 0.734 (0.685 to 0.783) | 2.46 (2.05 to 2.95) | 1.01 | 1.02 |
| Excluding 16 procedures from 2026 | 1,470 | 117 | 0.736 (0.687 to 0.785) | 2.52 (2.10 to 3.02) | 1.04 | 1.04 |
| **STS PROM** |  |  |  |  |  |  |
| Primary analysis (1,076 procedures) | 1,076 | 66 | 0.815 (0.757 to 0.873) | 2.72 (2.14 to 3.47) | 1.11 | 1.31 |
| Excluding the 2026 procedures | 1,063 | 66 | 0.815 (0.757 to 0.873) | 2.75 (2.16 to 3.50) | 1.12 | 1.31 |
| Isolated CABG only | 757 | 34 | 0.749 (0.657 to 0.842) | 2.65 (1.89 to 3.70) | 1.03 | 1.13 |

Multiple imputation: chained equations with 20 imputations (age, sex, creatinine, weight, ejection fraction, dialysis, NYHA class, shock, heart failure, urgency, weight of intervention, death, diabetes, endocarditis, critical state); the O/E ratio ranged from 2.42 to 2.45 across imputations, and the table shows the result for the averaged predictions. AUC, area under the receiver operating characteristic curve; CITL, calibration in the large; O/E, observed to expected ratio.

**Figure S1.** EuroSCORE II observed to expected ratio (95% confidence interval) under each sensitivity scenario. The dashed line indicates perfect calibration in the large.

# S5. Procedures outside the scope of the STS 2018 models

**Table S5.** Reasons why procedures were not evaluated with STS PROM

| **Reason** | **n** |
| --- | --- |
| No STS procedure or more than one recorded (includes combined aortic and mitral valve procedures and procedures without CABG or aortic or mitral valve surgery) | 154 |
| Concomitant procedure outside the STS models (ascending aorta, maze, septal defect closure, tumour resection, other) | 117 |
| STS procedure flag inconsistent with the EuroSCORE II weight of intervention | 98 |
| Surgery on the thoracic aorta (EuroSCORE II item) | 41 |
| **Total outside the STS scope** | **410** |

Reasons were applied in the order shown in the Methods; each procedure is counted once. The 1,076 eligible procedures comprised 757 isolated CABG, 186 isolated AVR, 54 isolated MVR, 51 AVR plus CABG, 26 MVR plus CABG, and 2 mitral valve repairs.

# S6. Precision, temporal validation and updating of EuroSCORE II, and altitude-related markers

## S6.1. Precision attainable with the study sample

To examine whether the discrepancy between good discrimination and poor calibration could reflect insufficient statistical power, we assessed the precision attainable with the cohort using the criteria of Riley et al. (2021) for external validation of a prediction model with a binary outcome. With 117 deaths among 1,486 procedures (outcome proportion 7.9%), a perfectly calibrated model would yield an O/E ratio with a 95% confidence interval of approximately 0.84 to 1.19. The observed EuroSCORE II O/E ratio of 2.50 (95% CI 2.09 to 3.00) lies far outside this range. With 47 expected deaths under EuroSCORE II, the study had 80% power (two-sided alpha 0.05) to detect an O/E ratio of 1.51 or more. Low power therefore cannot explain the observed miscalibration; if anything, limited power would bias the study towards not detecting miscalibration. Precision is limited for the calibration slope and for small subgroups (Table S6).

**Table S6.** Precision of validation measures attainable with the study sample

| **Measure** | **Precision with N=1,486 and 117 events** | **Common target** | **N needed for target** |
| --- | --- | --- | --- |
| O/E ratio | SE(ln O/E) 0.089; 95% CI for a true O/E of 1: 0.84 to 1.19 | CI from 0.9 to 1.1 | 4,496 |
| Calibration slope | SE 0.111; CI width 0.43 | CI width 0.2 | 7,016 |
| AUC (C statistic) | SE 0.024; CI width 0.094 | CI width 0.10 | 1,303 |
| Minimum detectable O/E ratio | 1.51 (80% power, 47 expected deaths) |  |  |

Calculated with the formulas of Riley et al. (2021), using the observed outcome proportion and, for the calibration slope, the distribution of the recalibrated EuroSCORE II linear predictor. The cohort exceeds the commonly recommended minimum of 100 events for external validation. AUC, area under the receiver operating characteristic curve; CI, confidence interval; O/E, observed to expected ratio; SE, standard error.

## S6.2. Temporal validation of updated EuroSCORE II

To examine whether local updating corrects the miscalibration, we derived updated versions of EuroSCORE II in one part of the cohort and tested them in the other. The cohort was split chronologically, which is a stronger test of transportability than a random split: procedures from 2019 to 2021 (n=735, 58 deaths) were used for updating and procedures from 2022 to February 2026 (n=751, 59 deaths) for validation. Three updating methods of increasing complexity were compared (Janssen et al., 2008): recalibration in the large (new intercept, estimated update 0.87 on the logit scale), logistic recalibration (new intercept 0.98 and slope 1.03, 95% CI 0.74 to 1.33), and model revision, in which all EuroSCORE II coefficients were re-estimated with ridge penalisation (penalty chosen by 5-fold cross-validation) to limit overfitting given the small number of events.

**Table S7.** Performance in the temporal validation period (2022 to 2026, n=751) of EuroSCORE II updated with 2019 to 2021 data

| **Model** | **AUC (95% CI)** | **O/E (95% CI)** | **CITL (95% CI)** | **Slope (95% CI)** | **Brier** | **ICI** | **HL p** |
| --- | --- | --- | --- | --- | --- | --- | --- |
| Original EuroSCORE II | 0.727 (0.653 to 0.802) | 2.97 (2.30 to 3.83) | 1.20 (0.93 to 1.48) | 1.16 (0.81 to 1.51) | 0.0713 | 0.053 | <0.001 |
| Recalibration in the large | 0.727 (0.653 to 0.802) | 1.33 (1.03 to 1.72) | 0.33 (0.05 to 0.60) | 1.16 (0.81 to 1.51) | 0.0669 | 0.020 | 0.029 |
| Logistic recalibration | 0.727 (0.653 to 0.802) | 1.35 (1.04 to 1.74) | 0.34 (0.06 to 0.61) | 1.12 (0.78 to 1.46) | 0.0669 | 0.021 | 0.027 |
| Model revision (all coefficients re-estimated, ridge) | 0.736 (0.661 to 0.811) | 1.20 (0.93 to 1.55) | 0.22 (-0.06 to 0.50) | 0.98 (0.70 to 1.26) | 0.0656 | 0.017 | 0.428 |

AUC, area under the receiver operating characteristic curve; CITL, calibration in the large; HL, Hosmer-Lemeshow; ICI, integrated calibration index; O/E, observed to expected ratio.

Updating the intercept alone reduced the O/E ratio from 2.97 to 1.33. The residual underestimation in the validation period reflects the higher crude mortality in 2022 rather than a failure of the method. Re-estimating all coefficients improved discrimination only slightly (AUC 0.736 versus 0.727). We also performed a random split, with 700 procedures for updating and the remaining 786 for testing, repeated 200 times (Table S8). With a random split, all three methods produced O/E ratios close to 1 in the test sets, as expected when development and test samples come from the same period.

**Table S8.** Random split (700 procedures for updating, 786 for testing), 200 repetitions

| **Model** | **AUC, median [IQR]** | **O/E, median [IQR]** | **Calibration slope, median [IQR]** |
| --- | --- | --- | --- |
| Original EuroSCORE II | 0.738 [0.724 to 0.752] | 2.50 [2.36 to 2.64] | 1.06 [0.98 to 1.11] |
| Recalibration in the large | 0.738 [0.724 to 0.752] | 1.00 [0.89 to 1.13] | 1.06 [0.98 to 1.11] |
| Logistic recalibration | 0.738 [0.724 to 0.752] | 1.00 [0.89 to 1.13] | 1.02 [0.87 to 1.14] |
| Model revision (all coefficients re-estimated, ridge) | 0.746 [0.731 to 0.762] | 1.00 [0.90 to 1.14] | 1.09 [0.96 to 1.42] |

IQR, interquartile range across the 200 random splits.

**Table S9.** Published and locally re-estimated EuroSCORE II coefficients

| **Term** | **Published EuroSCORE II β** | **Locally re-estimated β (ridge, 2019 to 2021)** |
| --- | --- | --- |
| Age (per year over 60) | 0.029 | 0.035 |
| Female sex | 0.220 | 0.508 |
| On dialysis | 0.642 | 1.787 |
| Creatinine clearance 50 mL/min or less | 0.859 | 0.088 |
| Creatinine clearance 51 to 85 mL/min | 0.304 | -0.219 |
| Extracardiac arteriopathy | 0.536 | -0.038 |
| Poor mobility | 0.241 | -0.450 |
| Previous cardiac surgery | 1.119 | 0.988 |
| Chronic lung disease | 0.189 | 0.333 |
| Active endocarditis | 0.619 | 1.446 |
| Critical preoperative state | 1.087 | 0.607 |
| Diabetes on insulin | 0.354 | 0.091 |
| CCS class 4 angina | 0.223 | 0.356 |
| Recent myocardial infarction | 0.153 | 0.141 |
| Surgery on thoracic aorta | 0.653 | 0.484 |
| NYHA class II | 0.107 | 0.020 |
| NYHA class III | 0.296 | 0.226 |
| NYHA class IV | 0.560 | 0.871 |
| LVEF 31% to 50% | 0.315 | 0.305 |
| LVEF 30% or less | 0.808 | 0.048 |
| PASP 31 to 55 mmHg | 0.179 | -0.088 |
| PASP over 55 mmHg | 0.349 | 0.612 |
| Urgent | 0.317 | 0.356 |
| Emergency or salvage | 0.704 | 1.999 |
| Single non-CABG procedure | 0.006 | 0.065 |
| Two procedures | 0.552 | 0.640 |
| Three or more procedures | 0.972 | 0.478 |

Re-estimation used 58 events for 27 terms and is therefore exploratory; ridge penalisation shrinks coefficients towards zero. The poor LV function term combines the poor and very poor categories (published coefficient shown for the poor category). CCS, Canadian Cardiovascular Society; LVEF, left ventricular ejection fraction; NYHA, New York Heart Association; PASP, pulmonary artery systolic pressure.

Terms with a clearly larger local weight were dialysis, emergency or salvage surgery, active endocarditis, and female sex, whereas moderately impaired creatinine clearance and poor left ventricular function carried less weight than in the published model. These differences suggest that, beyond a shift in baseline risk, some predictors may behave differently in our population, but the number of events is too small to support a new model.

**Figure S2.** Calibration in the temporal validation period (2022 to 2026) of the original EuroSCORE II and of the three updating methods derived from 2019 to 2021 data. Points show deciles of predicted risk with 95% confidence intervals; the solid line is a loess smoother; the dashed diagonal indicates perfect calibration.

## S6.3. Altitude-related physiological markers

Because every patient in this cohort was operated at the same altitude, altitude itself cannot be included as a predictor: its effect, if any, is absorbed into the model intercept together with case mix and local practice. What can be examined is whether physiological markers related to altitude add prognostic information to EuroSCORE II. We added preoperative haematocrit, haemoglobin, and echocardiographic pulmonary artery systolic pressure, one at a time, to a logistic model containing the EuroSCORE II linear predictor (Table S10).

**Table S10.** Altitude-related physiological markers added to EuroSCORE II

| **Marker added to EuroSCORE II** | **n / deaths** | **OR (95% CI)** | **p (linear term)** | **p (linear and quadratic)** | **AUC without to with marker** |
| --- | --- | --- | --- | --- | --- |
| Preoperative haematocrit (per 1%) | 1,347 / 105 | 0.93 (0.91 to 0.96) | <0.001 | <0.001 | 0.735 to 0.756 |
| Preoperative haemoglobin (per 1 g/dL) | 1,390 / 109 | 0.86 (0.79 to 0.94) | <0.001 | 0.004 | 0.738 to 0.753 |
| Echocardiographic PASP (per 10 mmHg) | 590 / 62 | 1.16 (0.98 to 1.37) | 0.083 | 0.163 | 0.759 to 0.770 |

Complete cases for each marker. The p value in the fifth column is from a likelihood ratio test of the linear and quadratic terms together. AUC, area under the receiver operating characteristic curve; OR, odds ratio; PASP, pulmonary artery systolic pressure.

Lower preoperative haematocrit and haemoglobin were associated with higher mortality beyond EuroSCORE II and increased the AUC by about 0.02, whereas pulmonary artery systolic pressure did not add significant information (60% of values were missing). Secondary erythrocytosis was rare (23 of 1,347 patients with a haematocrit measurement), so the excess risk is not explained by erythrocytosis. Anaemia defined with altitude-adjusted thresholds was common (635 of 1,390 patients with a haemoglobin measurement), which suggests that haemoglobin interpreted with altitude-specific cut-offs is a candidate predictor for a future high-altitude model. Developing and validating such a model will require multicentre data from several high-altitude centres and a larger number of events.

# References

Nashef SAM, Roques F, Sharples LD, Nilsson J, Smith C, Goldstone AR, et al. EuroSCORE II. Eur J Cardiothorac Surg. 2012;41: 734-745.

Nashef SA, Roques F, Michel P, Gauducheau E, Lemeshow S, Salamon R. European system for cardiac operative risk evaluation (EuroSCORE). Eur J Cardiothorac Surg. 1999;16: 9-13.

Riley RD, Debray TPA, Collins GS, Archer L, Ensor J, van Smeden M, et al. Minimum sample size for external validation of a clinical prediction model with a binary outcome. Stat Med. 2021;40: 4230-4247.

Janssen KJM, Moons KGM, Kalkman CJ, Grobbee DE, Vergouwe Y. Updating methods improved the performance of a clinical prediction model in new patients. J Clin Epidemiol. 2008;61: 76-86.

Semenkovich N. sts-risk-calculator-cli. GitHub; 2022. https://github.com/semenko/sts-risk-calculator-cli
